# Supplementary material for: Unravelling the genetic landscape of cervical insufficiency: Insights into connective tissue dysfunction and hormonal pathways
Source: PLoS One. 2024 Sep 19;19(9):e0310718. doi: 10.1371/journal.pone.0310718 (PMC11412527; doi:10.1371/journal.pone.0310718)
Supplement: S3 Table — (PDF) [file pone.0310718.s003.pdf]

Supplement 3

Connective tissue dysfunction assessment questionnaire, including Beighton and Brighton criteria

Question	Answer	Max score
Beighton criteria	0-9	9
**Brighton questions:**		
**Major criteria:** Beighton score ≥4	YES/NO	
**Major criteria:** Joint pain lasting >3 months in 1-3 joints or back pain >3 months or spondylitis, spondylolisthesis	YES/NO	1
**Minor riteria:**		
Dislocation of >1 joint or recurrent dislocations of the same joint	YES/NO	1
Arthritis of 3 or more joints (including epicondylitis, tenosynovitis, bursitis)	YES/NO	1
Marfanoid habitus (high, hypostenic, arms' length > height (>1.03), long thin fingers)	YES/NO	1
Very supple, thin/brittle skin, abnormal scarring	YES/NO	1
Ocular pathology (myopia, glaucoma, ocular ptosis, corneal damage)	YES/NO	1
Varicose veins, hernias, uterine prolapse, rectal prolapse, vascular aneurysms / ruptures	YES/NO	1
Cardiac valve pathology (MVN, AoVN)	YES/NO	1
Muscle pain lasting longer than 3 months	YES/NO	1
Scoliosis	YES/NO	1
Frequent or atypical bone fractures	YES/NO	1
Urinary incontinence	YES/NO	1
Frequent consultations with a rheumatologist	YES/NO	1
Family history indicative of connective tissue disorder in a first-degree relative	YES/NO	1
Have any of your first-degree relative (sister/mother) experienced repeated preterm births or late pregnancy losses?	YES/NO	1
Patient herself is born preterm (<37 weeks)	YES/NO	1
	**TOTAL:**	**25**

At least four Beighton criteria indicates of joint hypermobility. Requirements for the clinical diagnosis of Ehlers-Danlos syndrome according to Brighton includes any of the following:

1. Two major criteria
2. One major + two minor
3. Four minor criteria
4. Two minor criteria and unequivocally affected first-degree relative in a family history
