# Supplementary material for: Unravelling the genetic landscape of cervical insufficiency: Insights into connective tissue dysfunction and hormonal pathways
Source: PLoS One. 2024 Sep 19;19(9):e0310718. doi: 10.1371/journal.pone.0310718 (PMC11412527; doi:10.1371/journal.pone.0310718)
Supplement: Attachment — Submitted filename: Response to Reviewers.docx [file pone.0310718.s005.docx]

## Dear Burak Bayraktar

## Dear Reviewer,

## Thank you very much for processing and reviewing our manuscript. We have addressed all the comments and provided point-by-point answers below, incorporating the suggested changes into the manuscript.

## The provided line numbers are from the unmarked version of revised paper without tracked changes.

## Editor:

**Question:** “We conducted whole exome sequencing (WES) analysis on 114 patients diagnosed with a short cervix, CI or a history of CI in previous pregnancies.” How did you choose the cases? More precisely, was this study a prospective study on short cervix? Or were patients who underwent WES examination for other indications retrospectively investigated for short cervix?

**Answer:** The process of enrolling patients in the study is described in the Methods section (lines 483-502). Briefly, patients undergoing routine antenatal care were examined for a short cervix, and those requiring treatment for cervical shortening were recruited. This study was conducted as a prospective longitudinal cohort study, as detailed in the Methods section (lines 484).

**Question:** Can you include patient’s demographics? Smoking, uterine anomaly, systemic diseases, etc. was the excluded?

**Answer:** Some patient demographics, including ethnicity, age, and parity, are described in the Results section (lines 128-132). Additional phenotype data was collected using the connective tissue dysfunction assessment questionnaire, as detailed in the Methods section (lines 497-499). We have now supplemented the Methods section with the exclusion criteria, which also includes uterine anomalies. Notably, systemic diseases (unless part of a genetic syndrome) and smoking were not exclusion criteria.

**Question:** I guess it means you found no significant genome changes?

**Answer**: None of the patients in our study were found to have monogenic connective tissue disorder. Instead, our main finding was the identification of rare damaging variant enrichment in steroid hormone receptor pathway genes, with variants in *PGR*, *HSP90AA1*, and *NR3C1* reaching genome-wide statistical significance even after correction for multiple testing (as indicated in lines 262-263 of the Results section).

## Reviewer #1:

**Question:** Study of important and relevant themes for the magazine. The main objective of the study was to deepen discussions about the genetic factors that contribute to IC.

The study brings relevant results, but its biggest problem is that it does not present a methods session. There is a very brief paragraph in the discussion commenting on what was carried out in the study. I strongly suggest that the authors insert a methods session into the study involving study design, setting (where the study was carried out), population studied, inclusion and exclusion criteria, how these participants were recruited, procedures carried out for data collection, analysis of data, ethics committee, etc. Without this session, it is not possible to evaluate the study and say whether it was carried out correctly or whether there were biases.

**Answer:** Thank you sincerely for dedicating your time and effort to reviewing our manuscript. We apologize for any confusion regarding the Methods section's visibility. You can find the Methods section starting with line 474. The Methods placement after the Discussion is in line with the formatting guidelines stipulated by PLOS One.

**Question:** Another point that I think is important to mention is that the title is not eye-catching and does not arouse the reader's curiosity. I leave as a suggestion: Unraveling the Genetic Landscape of Cervical Insufficiency: Insights into Connective Tissue Dysfunction and Hormonal Pathways

**Answer**: Thank you very much for this nice suggestion, with pleasure we are accepting this new title.

**Question:** In the summary, I suggest clearly and objectively stating the objective of the study and also structuring a methods session.

**Answer**: The primary aim of our study is stated in lines 115-116, and we have revised it for clarity.

**Question:** Keywords: lots of keywords. Choose a maximum of five.

**Answer:** We understand the concern regarding the abundance of keywords. However, PLOS One encourages the inclusion of multiple keywords to enhance the discoverability of published articles. Therefore, we prefer to retain the current keywords.

**Question:** In the introduction, it is important to provide a little more detail about the reasons why the PTB is a public health concern. What are its implications?

**Answer**: We have briefly addressed the paragraph on PTB (please see lines 46-50). However, we chose not to significantly expand this paragraph as considerable literature already exists discussing the social and economic concerns of PTB, as referenced in this paragraph. Given that the primary focus of our article is the genetics of cervical insufficiency, we opted not to shift the primary focus of the study.

**Question:** Furthermore, what were the motivations for conducting the study?

**Answer**: Our primary motivation for studying the genetics of CI stems from clinical needs, as pregnancy outcomes in this patient group are particularly concerning (see lines 97-99 and 276-278 in the Discussion section). The genetics of PTB and particularly CI remain poorly understood, with existing literature often lacking clinical subgrouping. We aimed to address this gap by focusing specifically on the CI patient group, anticipating insights into specific genes and molecular pathways, particularly related to the connective tissue hypothesis (112-114).

**Question:** What are the hypotheses?

**Answer**: As you may understand, the hypothesis for this study stems from our previous pilot study where we identified variants in extracellular matrix-related genes in patients with cervical insufficiency. This led us to hypothesize that CI might represent a subtle form of connective tissue disorder (please refer to lines 108-114).

**Question:** What new does the study bring?

**Answer**: While we understand that some studies choose to highlight findings in the Introduction, we have opted to reserve the anticipation of study results for the Results and Discussion sections.

**Question:** What knowledge gaps do you aim to fill?

Our study aims to address significant knowledge gaps in the field of cervical insufficiency. Currently, there are no aetiological treatments available for this condition, largely due to limited understanding of its molecular mechanisms and the neglect of CI genetics in the scientific community. Our goal is to decipher the molecular pathways underlying CI to contribute to a better understanding of its pathogenesis.

**Question:** The justification for the study needs to be clearer and better defined in the introduction.

**Answer**: Thank you very much for the detailed analysis of our Introduction section and providing valuable insights. We have made substantial changes in the Introduction to better clarify the justification for conducting our study.

As outlined in the Introduction, there is limited understanding of the genetics of cervical insufficiency, and our pilot study was among the first to apply next-generation sequencing to this patient group, for the current study we collected the largest cohort of patients for whole exome sequencing. We aimed to validate the long-standing link between connective tissue dysfunction and CI through genetic studies and developed the connective tissue dysfunction assessment as a comprehensive clinical tool for this purpose.

Unexpectedly, our study also revealed significant findings related to the progesterone pathway, despite the modest sample size. We hope you will find this aspect of the study particularly intriguing.

**Question:** Regarding the results and discussion, I have no comments at first, I would need to know more details about how the study was conducted (methods) to be able to assess whether the results were reported and discussed appropriately.

**Answer**: Thank you for your initial assessment. We understand that a thorough evaluation of the Results and Discussion sections depends on a comprehensive understanding of the study methods. We hope that our Methods section will provide the necessary details, and we look forward to your feedback on the manuscript once it is complete.

Once again, we hope our clarifications have addressed all current concerns, and we remain open to any further suggestions or feedback you may have.

Thank you once again for your valuable input.

Kind regards,

Authors
