# Supplementary material for: Unravelling the genetic landscape of cervical insufficiency: Insights into connective tissue dysfunction and hormonal pathways
Source: PLoS One. 2024 Sep 19;19(9):e0310718. doi: 10.1371/journal.pone.0310718 (PMC11412527; doi:10.1371/journal.pone.0310718)
Supplement: Attachment — Submitted filename: Response to Reviewers_21_08_2024.docx [file pone.0310718.s006.docx]

## Dear Burak Bayraktar,

## Dear Reviewer,

## Thank you very much for processing and reviewing our manuscript. We have addressed all the comments and provided point-by-point answers below, incorporating the suggested changes into the manuscript.

## The provided line numbers are from the unmarked version of revised paper without tracked changes.

Reviewer #2:

Dear Authors,

this is an interesting prospective longitudinal cohort study, trying to identify the molecular causes of cervical insufficiency. This one represents one of the origins of the main cause itself of fetal morbidity and mortality in Obstetrics: Preterm Birth. I read with great interest the Manuscript, which falls within the aim of this Journal. It is the revision number two for this journal. Several of the corrections required have been applied. In my honest opinion, some major improvements and corrections are still needed.

Title- I see the corrections applied

Abstract:

The abstract should better be structured for sake of clarity. It should contain background, Objective, methods, Results and Conclusion. More insights regarding the methods are already needed

**Answer**: Dear Reviewer, thank you very much for your time and valuable feedback on improving our manuscript. We have restructured the Abstract to include Background, Methods, Results and Conclusion. We have enhanced the text to provide more details and clarity.

Introduction:

Improvements have been made according to the previous reviewer advices, but a descriptive paragraph of what preterm birth is, should be written. I have no objections for the rest of the introduction

**Answer:** Please refer to lines 48-79 of the Introduction, which includes a sub-section titled ‘Preterm birth: one outcome – multiple aetiologies and distinct molecular pathways’ (line 53) providing prevalence, etiological pathways, contributing factors, and current treatment options of PTB.

Results: This paragraph appears too repetitive and it should not just be the list of the collected data. Numbers should be presented in a systematic and goal-oriented manner.

**Answer:** We have revised the Results section to minimize repetition and present data systematically. The structure focuses on:

1. Variant analysis in genes associated with connective tissue disorders, based on ACGS guidelines, to explore the hypothesis that CI might reflect a subtle connective tissue disorder. We also correlated genetic findings with connective tissue dysfunction assessment results (lines 317-360).

2. Genome-wide (a hypothesis-free) rare variant burden test that identified *PGR*, *HSP90AA1*, and *NR3C1* genes as potentially significant in CI development (lines 361-417).

3. Pathway enrichment analyses performed both in a targeted manner and genome-wide (lines 418-433).

We believe our revisions address the concern about repetitiveness and present the data in a clear manner.

Discussion:

The discussion should elucidate and find possible explanations for the research. It should be revised in the light of the methods.

**Answer:** The Discussion section provides detailed explanations and contextualize our findings based on the methodologies used. The discussion is organized into sub-sections corresponding to the different perspectives of our analysis (i.e., connective tissue perspective, hormonal pathway perspective). Each sub-section addresses our findings in the context of existing literature and aims to elucidate possible explanations.

If Reviewer have specific concerns or inquiries regarding any part of the discussion, we would be happy to address them.

Conclusions:

Rename this section.

It should be more consistent and summarize your main findings, clinical implications and suggest areas for further research.

Avoid overgeneralization.

**Answer**: We have renamed and revised the Conclusions section to ensure it provides a clear and concise summary of our main findings, avoiding overgeneralization.

Materials and Methods:

There are some improvement compared to the first version, but is still not enough to make the manuscript scientifically sound. There is no chance to promote this kind of study lacking an appropriate Materials and Methods section.

**Answer**: The Methods section has now been moved after the Introduction and provides a detailed account of all steps performed during the study. If the Reviewer has specific concerns or requires further clarification on any aspect of this section, we would be happy to address them. We have aimed to ensure that all procedures involving patient and genomic data are thoroughly described.

Reviewer #3: Dear authors,

Thank you for revising the manuscript considering the previously suggestions from the reviewers in the first review round.

All the comments of the previously reviewers have been addressed by the authors and the manuscript was improved.

I strongly suggest to the authors to move the Methods, according to the Plos One submission guidelines called "Materials and Methods", after the Introduction section.

The "Materials and Methods" in the Plos One is located in the Middle section, after the introduction.

Please verify the manuscript organization located in this page: https://journals.plos.org/plosone/s/submission-guidelines

I do not have additional comments on the revision in this manuscript version.

**Answer**: Thank you very much for your time and efforts in reviewing our manuscript. We have moved the Methods section immediately after the Introduction.

Once again, we hope our clarifications have addressed all current concerns, and we remain open to any further suggestions or feedback you may have.

Thank you once again for your valuable input.

Kind regards,

Authors
